# Comparison of endoscopic healing and durability between infliximab originator and CT-P13 in pediatric patients with inflammatory bowel disease

**DOI:** 10.3389/fimmu.2024.1284181

**Published:** 2024-02-22

**Authors:** Eun Sil Kim, Sujin Choi, Byung-Ho Choe, Sowon Park, Yeoun Joo Lee, Sang Jun Sohn, Soon Chul Kim, Ki Soo Kang, Kunsong Lee, Jung Ok Shim, Yu Bin Kim, Suk Jin Hong, Yoo Min Lee, Hyun Jin Kim, So Yoon Choi, Ju Young Kim, Yoon Lee, Ji-Sook Park, Jae Young Kim, Dae Yong Yi, Ji Hyuk Lee, Kwang-Hae Choi, Hyo-Jeong Jang, In Sook Jeong, Ben Kang

**Affiliations:** ^1^ Department of Pediatrics, Kangbuk Samsung Hospital, Sungkyunkwan University School of Medicine, Seoul, Republic of Korea; ^2^ Department of Pediatrics, School of Medicine, Kyungpook National University, Daegu, Republic of Korea; ^3^ Crohn’s and Colitis Association in Daegu-Gyeongbuk (CCAiD), Daegu, Republic of Korea; ^4^ Division of Gastroenterology, Hepatology and Nutrition, Department of Pediatrics, Yonsei University College of Medicine, Severance Children’s Hospital, Seoul, Republic of Korea; ^5^ Department of Pediatrics, Pusan National University Children’s Hospital, Pusan National University School of Medicine, Yangsan, Republic of Korea; ^6^ Department of Pediatrics, Jeonbuk National University Hospital, Jeonbuk National University Medical School, Jeonju, Republic of Korea; ^7^ Department of Pediatrics, Jeju National University Hospital, Jeju, Republic of Korea; ^8^ Department of Pediatrics, Dankook University College of Medicine, Cheonan, Republic of Korea; ^9^ Department of Pediatrics, Korea University College of Medicine, Korea University Guro Hospital, Seoul, Republic of Korea; ^10^ Department of Pediatrics, Ajou University School of Medicine, Suwon, Republic of Korea; ^11^ Department of Pediatrics, Daegu Catholic University School of Medicine, Daegu, Republic of Korea; ^12^ Department of Pediatrics, Soonchunhyang University Bucheon Hospital, Soonchunhyang University College of Medicine, Bucheon, Republic of Korea; ^13^ Department of Pediatrics, Chungnam National University Hospital, Chungnam National University College of Medicine, Daejeon, Republic of Korea; ^14^ Department of Pediatrics, Kosin University Gospel Hospital, Kosin University College of Medicine, Busan, Republic of Korea; ^15^ Department of Pediatrics, Daejeon Eulji Medical Center, Eulji University, Daejeon, Republic of Korea; ^16^ Department of Pediatrics, Korea University Medical Center Anam Hospital, Seoul, Republic of Korea; ^17^ Department of Pediatrics, Gyeongsang National University College of Medicine, Jinju, Republic of Korea; ^18^ Institute of Medical Science, Gyeongsang National University, Jinju, Republic of Korea; ^19^ Department of Pediatrics, Gyeongsang National University Changwon Hospital, Changwon, Republic of Korea; ^20^ Department of Pediatrics, Chung-Ang University Hospital, Chung-Ang University, College of Medicine, Seoul, Republic of Korea; ^21^ Department of Pediatrics, Chungbuk National University College of Medicine, Chungju, Republic of Korea; ^22^ Department of Pediatrics, Yeungnam University School of Medicine, Daegu, Republic of Korea; ^23^ Department of Pediatrics, Keimyung University School of Medicine, Daegu, Republic of Korea; ^24^ Department of Pediatrics, Chung-Ang University, Gwangmyeong Hospital, Gwangmyeong, Republic of Korea

**Keywords:** children, inflammatory bowel disease, CT-P13, endoscopic healing, durability

## Abstract

**Background and aims:**

Favourable clinical data were published on the efficacy of CT-P13, the first biosimilar of infliximab (IFX), in pediatric inflammatory bowel disease (IBD); however, few studies have compared the effect on endoscopic healing (EH) and drug retention rate between the IFX originator and CT-P13. Therefore, we aimed to compare EH and the drug retention rate between the IFX originator and CT-P13.

**Methods:**

Children with Crohn’s disease (CD) and ulcerative colitis (UC)/IBD-unclassified (IBD-U) at 22 medical centers were enrolled, with a retrospective review conducted at 1-year and last follow-up. Clinical remission, EH and drug retention rate were evaluated.

**Results:**

We studied 416 pediatric patients with IBD: 77.4% had CD and 22.6% had UC/IBD-U. Among them, 255 (61.3%) received the IFX originator and 161 (38.7%) received CT-P13. No statistically significant differences were found between the IFX originator and CT-P13 in terms of corticosteroid-free remission and adverse events. At 1-year follow-up, EH rates were comparable between them (CD: *P*=0.902, UC: *P*=0.860). The estimated cumulative cessation rates were not significantly different between the two groups. In patients with CD, the drug retention rates were 66.1% in the IFX originator and 71.6% in the CT-P13 group at the maximum follow-up period (*P >*0.05). In patients with UC, the drug retention rates were 49.8% in the IFX originator and 56.3% in the CT-P13 group at the maximum follow-up period (*P >*0.05).

**Conclusions:**

The IFX originator and CT-P13 demonstrated comparable therapeutic response including EH, clinical remission, drug retention rate and safety in pediatric IBD.

## 1 Introduction

Inflammatory bowel disease (IBD) is a chronic gastrointestinal disorder, and the main subtypes include Crohn’s disease (CD) and ulcerative colitis (UC), with approximately 25% of cases diagnosed in children and adolescents (1). Pediatric-onset IBD typically manifests with a more severe phenotype than adult-onset IBD and is associated with an aggressive and complicated disease course (1, 2). The outcomes and prognosis of pediatric IBD significantly improved since the introduction of biologic agents as main therapeutic options approximately 20 years ago (3–6).

Owing to the severe and aggressive disease course, biologic agents are recommended for pediatric patients with IBD after risk stratification, and top–down therapy is recommended, particularly for patients with moderate-to-severe CD (7, 8). The first biologic agent introduced for the treatment of patients with IBD was infliximab (IFX), which targets tumour necrosis factor-α (TNF-α) and has significantly improved patient outcomes. Anti-TNF agents can induce clinical remission and endoscopic healing (EH), which is associated with a reduced need for hospitalization and surgery and, consequently, an improved quality of life (9, 10).

CT-P13 (Remsima, Celltrion, Incheon, South Korea; Inflectra, Pfizer, NY, USA) is the first biosimilar monoclonal antibody to reference IFX that is approved for use in all indications but is significantly more cost-efficient (11, 12). CT-P13 and IFX originator (Remicade, Janssen Biotech, Horsham) show comparable binding affinities to the monomeric and trimeric forms of human TNF-α and comparable TNF-α neutralising and cytotoxic activities (13). While preclinical comparative studies have confirmed a high degree of similarity and equivalent efficacy between the IFX originator and CT-P13 (14–19), concerns have been raised regarding drawing conclusions from rheumatoid arthritis and ankylosing spondylitis studies without direct clinical evidence in IBD (20).

Recently, favourable clinical data have been published on the efficacy of CT-P13 in pediatric IBD (15, 17, 21–25). However, these studies have not compared long-term clinical efficacy based on EH and durability between the IFX originator and CT-P13 in pediatric IBD. Therefore, we aimed to compare clinical outcomes including clinical remission, EH and drug retention rate of the IFX originator and CT-P13 based on data from a large observational cohort study of patients with CD and UC/IBD-unclassified (IBD-U).

## 2 Materials and methods

### 2.1 Patients and data collection

This retrospective observational study was conducted at the Department of Pediatrics of 22 centers in South Korea (see affiliations) between March 2015 and August 2022. Children and adolescents with IBD who had been treated with either the IFX originator or CT-P13 at age <19 years and followed for at least one year after initiation of IFX treatment were included. Patients who changed anti-TNF agents between IFX originator and CT-P13 and those with missing data on baseline clinicodemographics were excluded. CD, UC and IBD-U were diagnosed according to the revised Porto criteria of the European Society for Pediatric Gastroenterology, Hepatology and Nutrition (2). The choice between IFX originator and CT-P13 was made by patients and caregivers based on price and preference.

The study was approved by the Institutional Review Board of Kyungpook National University Chilgok Hospital and all other participating centers. Informed consent was waived because of the retrospective nature of the study.

Baseline clinicodemographics including sex, age at diagnosis and disease phenotype were collected at diagnosis, and the duration from diagnosis to IFX start, previous bowel and perianal surgery, previous biologic usage and concomitant immunomodulator were collected at IFX initiation. The disease phenotype at diagnosis was classified according to the Paris classification (26). Disease activity scores including the Pediatric Crohn’s Disease Activity Index (PCDAI) for CD and the Pediatric Ulcerative Colitis Activity Index (PUCAI) for UC and IBD-U were also collected, as well as laboratory findings, such as white blood cell (WBC) count, hemoglobin, platelet count, serum albumin, erythrocyte sedimentation rate (ESR), C-reactive protein (CRP) and fecal calprotectin (FC). The centers participating in this study are tertiary hospitals in South Korea, and each laboratory conducts total quality management to minimize inter-institutional errors and maintain test quality. Through this, thorough quality control is carried out to minimize errors in pre-analytical, analytical, and post-analytical stage, and SI units are used to ensure consistency of units.

Endoscopic scores including the Simple Endoscopic Score for Crohn’s Disease (SES-CD) for patients with CD and the endoscopic Mayo score for patients with UC were collected. Disease activity scores, laboratory findings and endoscopic scores were also collected during the 1-year treatment with IFX. The Mucosal Inflammation Non-invasive index (MINI) was also calculated at 1-year treatment with IFX (27). For CD patients, dose intensification with shortened IFX dosing intervals was allowed by Korean insurance coverage only if secondary loss of response (LOR) was suspected, i.e., if clinical symptoms worsened and CRP or FC levels increased significantly at two consecutive visits, necessitating dose intensification or a switch of therapy. All patients were treated according to the same treatment strategy at the time (28, 29). For the drug retention rate analysis, factors such as whether the patient was continuously on IFX and the maximum follow-up period while on IFX were also collected.

### 2.2 Endpoints, group allocation and definitions

The primary endpoint of this study was EH at 1-year treatment with IFX, and the secondary endpoint was corticosteroid-free remission (CSFR) 1 year after treatment with IFX and the drug retention rate during IFX treatment. The tertiary endpoint was adverse events during IFX treatment. Patients were divided into two groups according to whether they had received the IFX originator or CT-P13, and variables were compared separately between the groups among patients with CD and UC/IBD-U.

EH was defined as an SES-CD of <3 for patients with CD and an endoscopic Mayo score of <2 for patients with UC. CSFR was defined as a PCDAI of <10 in patients with CD and PUCAI of <10 in patients with UC while not requiring any treatment with corticosteroids. Drug retention rate was defined as probability of retention with IFX treatment after treatment initiation. Patients who stopped IFX and resumed it after more than 90 days were counted as permanent discontinuations.

### 2.3 Statistical analysis

For statistical comparisons between groups, Student’s *t* test and Wilcoxon’s rank-sum test were used for continuous variables and the chi-squared test or Fisher’s exact test for categorical variables. Comparative data for continuous variables were reported as medians with interquartile range (IQR) or means with standard deviation. To derive the estimated cumulative drug cessation rate during IFX treatment, Kaplan–Meier survival analysis was used. SAS version 9.4 (SAS Institute, Cary, NC, USA) was used for all statistical analyses.

## 3 Results

### 3.1 Baseline characteristics

**Figure 1:**
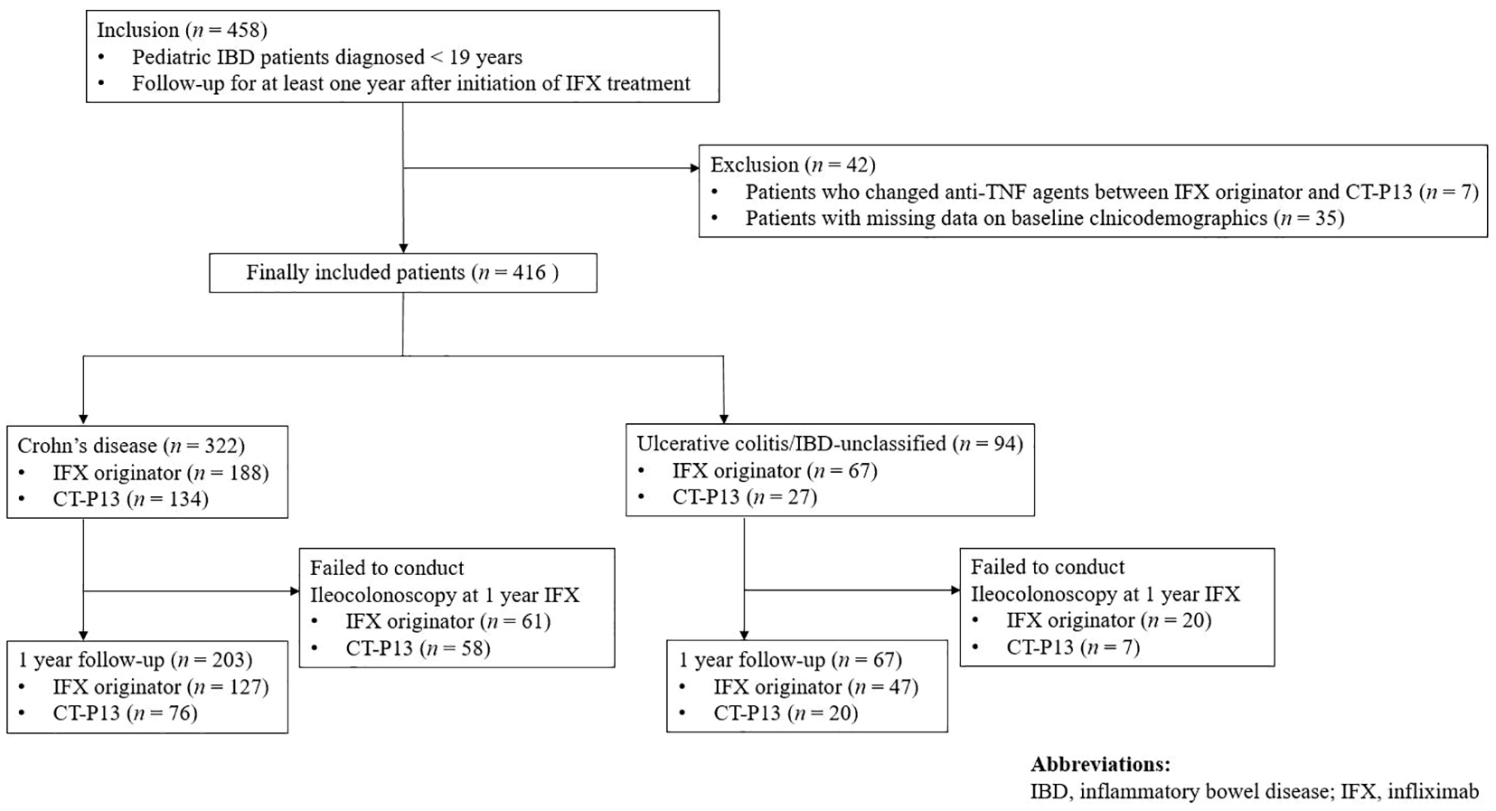
Flow diagram of the patient selection process. IBD, inflammatory bowel disease; IFX, infliximab.

In this study, a total of 458 patients were included. Forty-two patients who changed anti-TNF agents between IFX originator and CT-P13 and those with missing data on baseline clinicodemographics were excluded from the study, with 416 patients for final inclusion in the study (**Figure 1**). Among them, 322 (77.4%) patients were diagnosed with CD and 94 (22.6%) were diagnosed with UC/IBD-U. Men comprised 73.6% (237/322) of the CD group and 62.8% (59/94) of the UC/IBD-U group. Of the 322 patients with CD, 188 (58.4%) received IFX originator and 134 (41.6%) received CT-P13. Furthermore, 67 of the 94 patients with UC (71.3%) received IFX originator and 27 (28.7%) received CT-P13. In the comparison of baseline characteristics, no statistically significant difference was found between the IFX originator group and the CT-P13 group in neither patient with CD (**Table 1**) nor those with UC/IBD-U (**Table 2**).

**Table 1:** Baseline characteristics of patients with CD (*n* = 322).

	IFX originator (*n* = 188)	CT-P13 (*n* = 134)	*P*
At diagnosis			
Male sex, *n* (%)	139 (73.9)	98 (73.1)	0.974
Age at diagnosis (years), median (IQR)	14.0 (12.0–15.8)	13.8 (11.8–15.9)	0.675
Paris – Age, *n* (%)			0.973
A1a	13 (6.9)	10 (7.5)	
A1b	152 (80.9)	107 (79.8)	
A2	23 (12.2)	17 (12.7)	
Paris – Lower GI tract involvement, *n* (%)			0.373
L1	19 (10.1)	17 (12.7)	
L2	22 (11.7)	11 (8.2)	
L3	146 (77.7)	103 (76.9)	
None	1 (0.5)	3 (2.2)	
Paris – Upper GI tract involvement, *n* (%)			0.384
None	52 (27.7)	46 (34.3)	
L4a	60 (31.9)	46 (34.3)	
L4b	32 (17.0)	18 (13.4)	
L4a + b	44 (23.4)	24 (17.9)	
Paris – Disease behavior, *n* (%)			0.365
B1	146 (77.7)	108 (80.6)	
B2	26 (13.8)	20 (14.9)	
B3	16 (8.5)	6 (4.5)	
Paris – Perianal disease modifier, *n* (%)			0.737
No	72 (38.3)	48 (35.8)	
Yes	116 (61.7)	86 (64.2)	
Paris – Growth			0.319
G0	124 (69.7)	101 (75.4)	
G1	57 (30.3)	33 (24.6)	
At IFX initiation			
Duration from diagnosis to IFX (years), median (IQR)	0.27 (0.11–0.71)	0.23 (0.08–0.52)	0.109
IFX initiation within 3 months of diagnosis, *n* (%)	88 (46.8)	74 (55.2)	0.169
Previous bowel surgery, *n* (%)	1 (0.5)	2 (1.5)	0.573
Previous perianal surgery, *n* (%)	77 (41.0)	47 (35.1)	0.341
Previous biologic usage, *n* (%)	5 (2.7)	3 (2.2)	1.000
Concomitant IMM, *n* (%)	144 (76.6)	113 (84.3)	0.118
PCDAI, median (IQR)	40 (35–47.5)	37.5 (35–45)	0.117
WBC (/µL), median (IQR)	7745 (6270–10100)	7410 (6230–9220)	0.263
Hemoglobin (g/dL), median (IQR)	12.0 (10.9–13.2)	12.2 (11.2–13.0)	0.992
Platelet count (×10^3^/μL), median (IQR)	366 (293–456)	360 (309–429)	0.868
Albumin (g/dL), median (IQR)	4.1 (3.6–4.4)	4.1 (3.8–4.4)	0.301
ESR (mm/h), median (IQR)	32 (14–54)	30 (14–45)	0.212
CRP (mg/dL), median (IQR)	0.78 (0.47–2.5)	0.69 (0.45–1.39)	0.194
FC (mg/kg), median (IQR)	1665 (609–2725)	1415 (757–2325)	0.661
SES-CD, median (IQR)	12 (8–20)	13 (8–17)	0.285

B1, non-structuring non-penetrating behavior; B2, structuring behavior; B3, penetrating behavior; CD, Crohn’s disease; CRP, C-reactive protein; FC, fecal calprotectin; ESR, erythrocyte sedimentation rate; GI, gastrointestinal; IFX, infliximab; IMM, immunomodulator; IQR, interquartile range; L1, distal 1/3 ileum ± limited caecal disease; L2, colonic disease; L3, ileocolonic disease; L4a + b, upper disease involvement in both the L4a and L4b; L4a, upper disease proximal to the ligament of Treitz; L4b, upper disease distal to the ligament of Treitz and proximal to the distal 1/3 ileum; PCDAI, Pediatric Crohn’s Disease Activity Index; SES-CD, Simple Endoscopic Score for Crohn’s Disease WBC, white blood cell count.

**Table 2:** Baseline characteristics of patients with UC/IBD-U (*n* = 94).

	IFX originator (*n* = 67)	CT-P13 (*n* = 27)	*P*
At diagnosis			
Male sex, *n* (%)	42 (62.7)	17 (63.0)	1.000
Age at diagnosis (years), median (IQR)	14.0 (12.9–15.8)	14.4 (11.1–15.8)	0.957
Paris – Extent, *n* (%)			0.447
E1	4 (6.0)	3 (11.1)	
E2	10 (14.9)	3 (11.1)	
E3	10 (14.9)	7 (25.9)	
E4	43 (64.2)	14 (51.8)	
Paris – Severity, *n* (%)			1.000
S0	52 (77.6)	21 (77.8)	
S1	15 (22.4)	6 (22.2)	
At IFX initiation			
Duration from diagnosis to IFX (years), median (IQR)	0.73 (0.34–1.38)	0.75 (0.41–1.97)	0.356
Previous bowel surgery, *n* (%)	1 (1.5)	1 (3.7)	0.494
Previous biologic usage, *n* (%)	1 (1.5)	0 (0)	1.000
Concomitant IMM, *n* (%)	40 (59.7)	20 (74.1)	0.282
Concomitant 5-ASA, *n* (%)	38 (56.7)	18 (66.7)	0.511
PUCAI, mean ± SD	59.0 ± 13.2	54.6 ± 11.8	0.138
WBC (/µL), median (IQR)	7400 (5555–11100)	7000 (5500–9160)	0.506
Hemoglobin (g/dL), mean ± SD	11.9 ± 2.2	11.6 ± 2.0	0.545
Platelet count (×10^3^/μL), median (IQR)	344 (284–525)	367 (272–658)	0.432
Albumin (g/dL), median (IQR)	4.1 (3.4–4.5)	4.3 (3.9–4.6)	0.086
ESR (mm/h), median (IQR)	23 (9–36)	12 (4–34)	0.144
CRP (mg/dL), median (IQR)	0.31 (0.06–1.35)	0.23 (0.08–1.39)	0.973
FC (mg/kg), median (IQR)	1924 (800–2127)	1800 (748–2398)	0.703
Endoscopic Mayo, *n* (%)			1.000
1	2 (5.5)	1 (5.3)	
2	10 (27.8)	5 (26.3)	
3	24 (66.7)	13 (68.4)	

CRP, C-reactive protein; E1, ulcerative proctitis; E2, left-sided ulcerative colitis (distal to the splenic flexure); E3, extensive (hepatic flexure distally); E4, pancolitis (proximal to the hepatic flexure); ESR, erythrocyte sedimentation rate; FC, fecal calprotectin; IBD-U, inflammatory bowel disease-unclassified; IFX, infliximab; IQR, interquartile range; PUCAI, Pediatric Ulcerative Colitis Activity Index; S0, never severe; S1, ever severe; SD, standard deviation; WBC, white blood cell count; UC, ulcerative colitis.

### 3.2 Comparison of outcomes at 1-year follow-up between the IFX originator and CT-P13 groups

**Figure 2:**
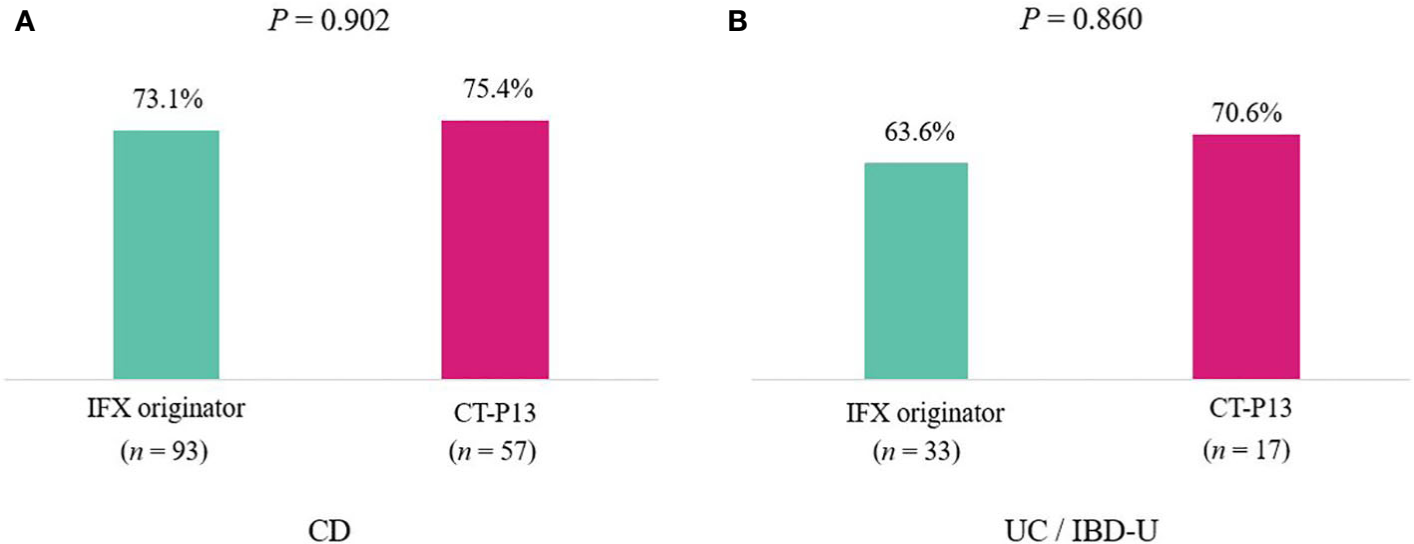
Endoscopic healing rates after 1 year of treatment with infliximab among **(A)** patients with CD (*n* = 150) and **(B)** patients with UC (*n* = 50). CD, Crohn’s disease; IBD-U, inflammatory bowel disease-unclassified; IFX, infliximab; UC, ulcerative colitis.

A comparison of outcomes at 1-year follow-up between the IFX originator and CT-P13 groups did not demonstrate any statistically significant difference among patients with CD (**Table 3**), including patients with UC/IBD-U (**Table 4**) . EH rates at 1-year follow-up were comparable between the two groups among patients with CD, with 73.1% (68/93) and 75.4% (43/57) of the IFX originator and CT-P13 groups achieving EH, respectively (*P* = 0.902) (**Figure 2A**). EH rates at 1-year follow-up were also comparable between the two groups among patients with UC/IBD-U, with 63.6% (21/33) and 70.6% (12/17) of the IFX originator and CT-P13 groups achieving EH, respectively (*P* = 0.860) (**Figure 2B**).

**Table 3:** Outcomes at 1-year follow-up in patients with CD (*n* = 203).

	IFX originator (*n* = 127)	CT-P13 (*n* = 76)	*P*
PCDAI, median (IQR)	0 (0–5)	0 (0–5)	0.823
CSFR, *n* (%)	112 (88.2)	68 (89.5)	0.960
WBC (/µL), median (IQR)	6170 (5010–7270)	5920 (5300–7100)	0.927
Hemoglobin (g/dL), median (IQR)	13.7 (12.6–14.7)	13.8 (12.8–14.7)	0.554
Platelet count (×10^3^/μL), median (IQR)	277 (241–339)	278 (235–325)	0.959
Albumin (g/dL), median (IQR)	4.4 (4.2–4.6)	4.4 (4.3–4.6)	0.942
ESR (mm/h), median (IQR)	9 (3–20)	9 (3–16)	0.545
CRP (mg/dL), median (IQR)	0.07 (0.04–0.34)	0.06 (0.04–0.15)	0.199
FC (mg/kg), median (IQR)	208 (50–706)	120 (52–446)	0.325
MINI, median (IQR)	5 (0–11)	5 (0–8)	0.461
SES-CD, median (IQR)	1 (0–3)	0 (0–2)	0.692

CD, Crohn’s disease; CRP, C-reactive protein; CSFR, corticosteroid-free remission; ESR, erythrocyte sedimentation rate; FC, fecal calprotectin; IFX, infliximab; IQR, interquartile range; MINI, Mucosal Inflammation Non-invasive index; PCDAI, Pediatric Crohn’s Disease Activity Index; SES-CD, Simple Endoscopic Score for Crohn’s Disease; WBC, white blood cell count.

**Table 4:** Outcomes at 1-year follow-up in patients with UC/IBD-U (*n* = 67).

	IFX originator (*n* = 47)	CT-P13 (*n* = 20)	*P*
PUCAI, median (IQR)	5 (0–10)	0 (0–10)	0.374
CSFR, *n* (%)	32 (68)	15 (75)	0.784
WBC (/µL), median (IQR)	6700 (5605–8925)	7030 (5815–8015)	0.924
Hemoglobin (g/dl), mean ± SD	13.0 ± 1.8	13.3 ± 1.7	0.542
Platelet count (×10^3^/μL), median (IQR)	291 (232–351)	325 (267–355)	0.212
Albumin (g/dL), mean ± SD	4.3 ± 0.4	4.5 ± 0.3	0.152
ESR (mm/h), median (IQR)	11 (3–23)	8 (4–21)	0.806
CRP (mg/dL), median (IQR)	0.06 (0.04–0.21)	0.04 (0.03–0.10)	0.075
FC (mg/kg), median (IQR)	574 (118–1350)	582 (312–1381)	0.583
Endoscopic Mayo, *n* (%)			0.273
0	9 (27.3)	10 (58.8)	
1	12 (36.3)	2 (11.8)	
2	7 (21.2)	3 (17.6)	
3	5 (15.2)	2 (11.8)	

CRP, C-reactive protein; CSFR, corticosteroid-free remission; ESR, erythrocyte sedimentation rate; FC, fecal calprotectin; IBD-U, inflammatory bowel disease-unclassified; IFX, infliximab; IQR, interquartile range; PUCAI, Pediatric Ulcerative Colitis Activity Index; SD, standard deviation; UC, ulcerative colitis; WBC, white blood cell count.

### 3.3 Comparison of drug retention rate between the IFX originator and the CT-P13 group

**Figure 3:**
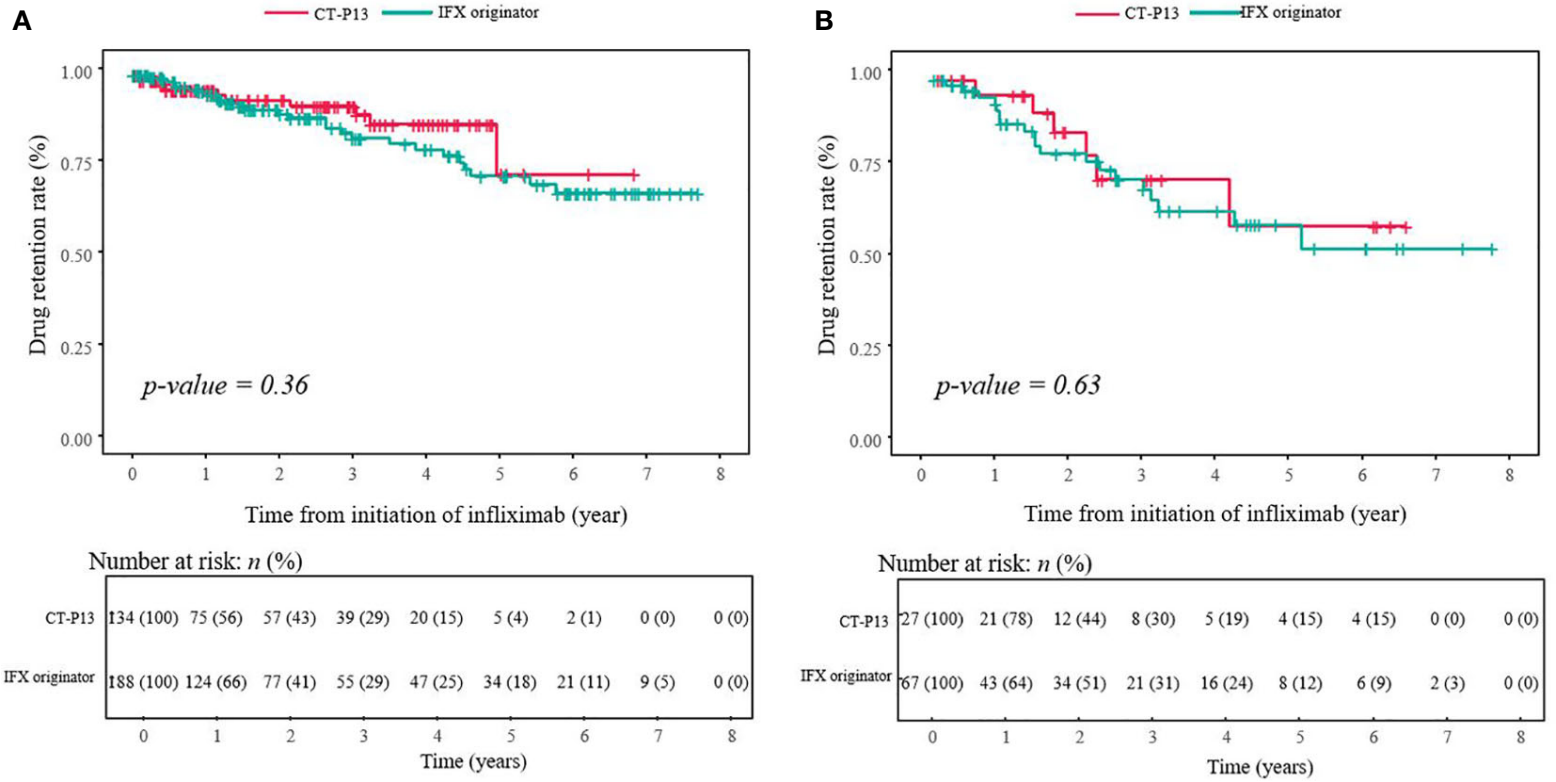
Drug retention curve during treatment with infliximab among **(A)** patients with CD (*n* = 322) and **(B)** patients with UC (*n* = 94). CD, Crohn’s disease; IBD-U, inflammatory bowel disease-unclassified; IFX, infliximab; UC, ulcerative colitis.

The median follow-up time for all patients with CD was 1.44 (range, 0.02–7.70) years, with 1.50 years in the IFX originator group and 1.22 years in the CT-P13 group (*P* = 0.20). After a median follow-up period of 1.44 years in patients with CD, approximately 10.2% (33/322) of patients discontinued treatment with IFX. According to the Kaplan–Meier survival analysis, the 1-, 2- and 5-year estimated cumulative cessation rates were 6.3%, 11.1% and 31.3% for the IFX originator group and 5.6%, 8.7% and 21.4% for the CT-P13 group, respectively. Moreover, the drug retention rates were 66.1% at the maximum follow-up period of 5.8 years in the IFX originator group and 71.6% at 5.0 years in the CT-P13 group. No statistical significance was found between the IFX originator and CT-P13 groups (*P* = 0.36) (**Figure 3A**).

After a median follow-up period of 1.85 years, 24.5% (23/94) of patients with UC/IBD-U discontinued treatment with IFX. The median follow-up time for all patients with UC was 1.85 (range, 0.02–7.87) years with 2.01 years in the IFX originator group and 1.84 years in the CT-P13 group (P = 0.97). After a median follow-up period of 1.84 years, 23.4% (22/94) of patients with UC/IBD-U discontinued treatment with IFX. According to the Kaplan–Meier survival analysis, the estimated cumulative cessation rates at 1, 2 and 5 years were 15.2%, 24.3% and 50.2% for the IFX originator group and 9.8%, 22.5% and 43.7% for the CT-P13 group, respectively. In addition, the drug retention rates were 49.8% at the maximum follow-up period of 5.2 years in the IFX originator group and 56.3% at 4.9 years in the CT-P13 group. No statistical significance was observed between the IFX originator and CT-P13 group (*P* = 0.63) (**Figure 3B**).

### 3.4 Comparison of adverse events between the IFX originator and CT-P13 groups

In total, 105/255 (41.2%) and 70/161 (43.5%) adverse events occurred in the IFX originator and CT-P13 groups, respectively, but with no statistical significance (*P* = 0.718). Infusion reactions occurred in 13/255 (5.1%) and 4/161 (2.5%) of the IFX originator and CT-P13 groups, respectively (*P* = 0.290) (**Table 5**).

**Table 5:** Adverse events during treatment with infliximab (*n* = 416).

	IFX originator (*n* = 255)	CT-P13 (*n* = 161)	*P*
Any adverse event, *n* (%)	105 (41.2)	70 (43.5)	0.718
Serious adverse event requiring inpatient hospitalization, *n* (%)	4 (1.6)	5 (3.1)	0.317
Infusion reaction, *n* (%)	13 (5.1)	4 (2.5)	0.290
Aggravation of previous bowel stenosis or penetration, *n* (%)	9 (3.5)	4 (2.5)	0.759
Newly developed bowel stenosis or penetration, *n* (%)	2 (0.8)	0 (0)	0.690
Aggravation of previous perianal fistulising disease, *n* (%)	5 (2.0)	6 (3.7)	0.436
Newly developed perianal fistulising disease, *n* (%)	2 (0.8)	1 (0.6)	1.000
Upper respiratory tract infection, *n* (%)	56 (22.0)	41 (25.5)	0.481
Sinusitis, *n* (%)	2 (0.8)	0 (0)	0.690
Otitis media/externa, *n* (%)	2 (0.8)	2 (1.2)	1.000
TB reactivation, *n* (%)	1 (0.4)	1 (0.6)	0.687
*de novo* TB, *n* (%)	0(0)	1 (0.6)	0.816
Herpes zoster reactivation, *n* (%)	2 (0.8)	2 (1.2)	1.000
EBV infection, *n* (%)	1 (0.4)	0 (0)	1.000
Norovirus infection, *n* (%)	0 (0)	2 (1.2)	0.291
Skin rash, *n* (%)	19 (7.5)	12 (7.5)	1.000
Acne, *n* (%)	10 (3.9)	7 (4.3)	1.000
Psoriasis, *n* (%)	4 (1.6)	0 (0)	0.280
Hidradenitis suppurativa, *n* (%)	2 (0.8)	1 (0.6)	1.000
Headache, *n* (%)	3 (1.2)	6 (3.7)	0.163
Hair loss, *n* (%)	8 (3.1)	2 (1.2)	0.368
Leukopenia, *n* (%)	10 (3.9)	4 (2.5)	0.608
Abnormal liver function tests, *n* (%)	10 (3.9)	9 (5.6)	0.580
Abnormal pancreas function tests, *n* (%)	3 (1.2)	7 (4.3)	0.084
Conjunctivitis, *n* (%)	2 (0.8)	2 (1.2)	1.000
Anterior blepharitis, *n* (%)	1 (0.4)	0 (0)	1.000
Osteoporosis, *n* (%)	1 (0.4)	1 (0.6)	1.000
Arthralgia/arthritis, *n* (%)	6 (2.4)	3 (1.9)	1.000
Peripheral neuropathy, *n* (%)	1 (0.4)	1 (0.6)	1.000
IgA nephropathy, *n* (%)	2 (0.8)	1 (0.6)	1.000
Polycystic ovary syndrome, *n* (%)	0 (0)	1 (0.6)	0.816

EBV, Epstein-Barr virus; IgA, immunoglobulin A; IFX, infliximab; TB, tuberculosis.

Serious adverse events requiring inpatient hospitalization was documented in 4/255 (1.6%) and 5/161 (3.1%) of the IFX originator and CT-P13 groups, respectively (*P* = 0.317). These included gastrointestinal obstructive signs and symptoms in five patients, massive gastrointestinal tract bleeding in two, tuberculosis reactivation in one, and *de novo* tuberculosis in one. No other serious adverse events were observed including life-threatening complications, resulting in death, persistent or significant disability/incapacity or congenital anomaly/birth defect.

## 4 Discussion

This multicenter study retrospectively evaluated and compared the clinical outcomes between the IFX originator and CT-P13 in pediatric patients with IBD. To the best of our knowledge, this is the first study to demonstrate equivalent EH achievement, IFX durability and drug retention rate between IFX originator and CT-P13 in a large and real-world pediatric IBD cohort. No significant differences were found in clinical remission, CSFR and biochemical remission between the IFX originator and CT-P13 groups (*P* > 0.05). In addition, a significant proportion of patients with CD and UC/IBD-U achieved EH with no difference between the two arms. Our results confirmed that CT-P13 was well tolerated and had a similar long-term efficacy as the IFX originator in patients with pediatric IBD.

Since the CT-P13 approval was based on the PLANETRA clinical trial results conducted in patients with rheumatoid arthritis and ankylosing spondylitis (30, 31), a steady stream of studies has been observed, which published on interchangeability between the IFX originator and CT-P13 in IBD (14, 16, 18, 19, 32). Furthermore, interest in the clinical use of CT-P13 is growing, which is expected to provide a similar clinical efficacy to IFX at a significant savings in terms of cost (33, 34). However, while a generic drug is an atomically identical copy of a reference drug, a biosimilar may not be completely identical to the originator biologic because a biologic agent is a structurally complex protein produced *in vivo* (20, 35). Even small changes in the cell line or laboratory conditions may result in minimal variations and differences from the reference drug. Therefore, to promote the appropriate use of CT-P13, a biosimilar of IFX, data on long-term clinical outcomes of CT-P13 in the treatment of IBD are needed. However, in pediatric patients, data on direct comparison of the clinical outcomes of the IFX originator and CT-P13 in the treatment of IBD were limited (15, 21).

To date, international guidelines indicate that CT-P13 is equivalent to the IFX originator in both adult and pediatric patients with IBD (36, 37). The interchangeability of CT-P13 with its originator has been well-established in both adult and pediatric patients with IBD (38–41). A prospective, open-label study of 143 adult patients with IBD (99 with CD, 44 with UC) who switched from the IFX originator to CT-P13 found that approximately 97% of patients remained on medication 6 months after switching, with few adverse events (38). The study also did not find differences in the disease activity, inflammatory marker levels and trough levels of IFX when comparing 6 months before and 6 months after switching from IFX originator to CT-P13. In line with the results of adult studies, several studies in pediatric patients with IBD have also demonstrated the interchangeability between the IFX originator and CT-P13 (15, 24, 42, 43). Kang et al. reported no statistically significant differences in disease activity, pharmacokinetics, immunogenicity and adverse events between the time of switching of the two drugs and during the 1-year follow-up (15).

To date, only two studies have directly compared the clinical efficacy and safety of IFX originator and CT-P13. A prospective study by Chanchlani et al., who analysed 82 children with IBD (63 with CD and 19 with UC/IBD-U), documented the lack of difference in the clinical remission rates on week 12 between the IFX originator group (68%) and the CT-P13 group (79%) (23). Moreover, Nikkonen et al. reported that 65% and 61% of the IFX originator and CT-P13 groups achieved clinical remission at 1 year, respectively (*P* > 0.05). In line with previous studies, the present study did not find significant differences in the disease activity scores and inflammatory marker levels at 1-year follow-up. In addition, our study showed that a significant proportion of pediatric patients with IBD maintained their CSFR after 1 year of treatment (88.6% (180/203) of patients with CD and 70.1% (47/67) of patients with UC), with no difference between the IFX originator and CT-P13-treated groups. Our results support that CT-P13 has therapeutic efficacy equivalent to the IFX originator in pediatric patients with IBD.

With an expanding array of therapeutic options, the goal of IBD treatment has shifted from symptom control to EH (7, 44). In the Selecting Therapeutic Targets in Inflammatory Bowel Disease-II study, EH is accepted as a long-term prognostic target for IBD treatment and reduces the risk of bowel damage (44, 45). Therefore, the difference in achieving EH and clinical remission in patients with IBD treated with IFX originator and CT-P13 must be evaluated. This study is significant in that it is the first to demonstrate the lack of differences between the two agents in achieving EH and drug retention rate in pediatric patients with IBD.

Current available literature data revealed no substantial difference in trough levels between the IFX originator and CT-P13 groups (15, 24, 43, 46). Serum IFX trough concentration is an important factor that influences the achievement of EH (odds ratio 1.479, 95% confidence interval 1.176–1.860, *P* < 0.001). In the light of these results, the lack of difference in EH achievement at 1 year of treatment between the IFX originator and CT-P13 arms is consistent. In the present study, EH was achieved in 73.1% of the patients in the IFX originator vs. 75.4% in the CT-P13 group (*P* = 0.902) in patients with CD and 63.6% in the IFX originator vs. 70.6% in the CT-P13 group (*P* = 0.860) in patients with UC/IBD-U. In addition, the MINI index was proposed by Martinus et al., a tool for the non-invasive assessment of mucosal inflammation in pediatric patients with CD using stool, FC, ESR and CRP (27). The study demonstrated that 78% of patients with MINI index scores <8 achieved EH, and scores of <6 had a positive predictive value of 86% for EH. In the CD cohort of our study, both the IFX and CT-P13 groups had a median MINI index of 5 at 1 year, with no difference between the two groups. This study was the first to demonstrate the lack of difference in the MINI index between the IFX originator and CT-P13 groups.

In the present study, CT-P13 is not inferior to the IFX originator in terms of IFX durability. According to the Kaplan–Meier curve, no significant difference was found in the estimated cumulative cessation rate between the IFX originator and CT-P13 groups. The durability rates of IFX were 88.2% at a median follow-up of 1.4 years in CD and 73.4% at a median follow-up of 1.85 years in UC. In patients with CD, the drug retention rates were 66.1% in the IFX originator and 71.6% in the CT-P13 group at the maximum follow-up period (*P* > 0.05). In patients with UC, the drug retention rate was 49.8% in the IFX originator and 56.3% in the CT-P13 group at the maximum follow-up period (*P* > 0.05). In addition, the proportions of patients with CD who remained on IFX maintenance therapy for 1 year were 93.7% in the IFX originator group and 94.4% in the CT-P13 group, and 84.8% and 90.2%, respectively, in patients with UC. A previous study in pediatric patients with IBD reported no statistically significant difference in the proportion of patients on maintenance therapy at 1 year: 65% for the IFX originator and 61.1% for the CT-P13 group (46).

The retention rate of treatment with biologic agents, or the probability of treatment persistence with the same biologic agents provides an index of overall drug effectiveness, patient satisfaction and treatment compliance. Our results were consistent with those of a previous study, revealing no difference in IFX drug retention rate between the IFX originator and CT-P13 groups (*P* > 0.05); however, the IFX durability rate was higher in our study than in the previous study. One possible explanation for the discrepancy between the results of the present study and of the former study is the higher rate of combination therapy with immunomodulators in the present study (47–50). In the present study, the proportions of combination therapy were 72.2% (184/255) in the IFX originator group and 82.6% (133/161) in the CT-P13 group, compared with 34.8% (8/23) and 53.6% (15/28), respectively, in the previous study. Furthermore, a large proportion of the patients in the present study had an early initiation of IFX strategy (49). In the present study, the median times from diagnosis to IFX initiation were 0.27 years in the IFX originator group and 0.23 years in the CT-P13 group for patients with CD and 0.77 years and 0.73 years for patients with UC, respectively.

The retrospective design of this study is a major limitation because it introduces the potential for confounding factors and bias that may affect the study results. However, all patients visited the outpatient clinic at regular intervals, allowing for a consistent clinical assessment of the therapeutic effects and adverse event monitoring. Second, laboratory results for trough concentrations and immunogenicity, which renders it possible to predict clinical outcomes and identify the cause of insufficient clinical response, were lacking. However, as mentioned above, previous studies have demonstrated the statistical similarity of the pharmacokinetics of the IFX originator and CT-P13; thus, it is unlikely to have affected our results. Third, our data were hospital-based rather than population-based; therefore, patients with a higher severity were included in the cohort, and a referral center bias cannot be excluded. However, most pediatric patients with IBD in Korea are referred to IBD specialists at university hospitals. Therefore, our data may be representative of the characteristics of pediatric patients with IBD in Korea. Despite these limitations, our data provide meaningful information that reflects the experience of long-term treatment with IFX originator and CT-P13 in a large, real-world cohort of pediatric patients with IBD.

In conclusion, the IFX originator and its biosimilar CT-P13 exhibited comparable therapeutic response including EH and clinical remission, durability and safety in pediatric patients with IBD. Our results are significant in that this is the first study to directly compare clinical outcomes, including EH at 1 year after treatment in pediatric patients with IBD treated with an IFX originator and CT-P13.

## Data availability statement

The original contributions presented in the study are included in the article/supplementary material. Further inquiries can be directed to the corresponding author.

## Ethics statement

The studies involving humans were approved by Institutional Review Board of Kyungpook National University Chilgok Hospital and all other participating centers. The studies were conducted in accordance with the local legislation and institutional requirements. Written informed consent for participation was not required from the participants or the participants’ legal guardians/next of kin in accordance with the national legislation and institutional requirements.

## Author contributions

ESK: Data curation, Formal analysis, Investigation, Visualization, Writing – original draft. SC: Data curation, Formal analysis, Investigation, Writing – original draft. B-HC: Data curation, Writing – review & editing. SP: Data curation, Writing – review & editing. YJL: Data curation, Writing – review & editing. SJS: Data curation, Writing – review & editing. SCK: Data curation, Writing – review & editing. KSK: Data curation, Writing – review & editing. KL: Data curation, Writing – review & editing. JOS: Data curation, Writing – review & editing. YBK: Data curation, Writing – review & editing. SJH: Data curation, Writing – review & editing. YML: Data curation, Writing – review & editing. HJK: Data curation, Writing – review & editing. SYC: Data curation, Writing – review & editing. JuYK: Data curation, Writing – review & editing. YL: Data curation, Writing – review & editing. J-SP: Data curation, Writing – review & editing. JaYK: Data curation, Writing – review & editing. DYY: Data curation, Writing – review & editing. JHL: Data curation, Writing – review & editing. K-HC: Data curation, Writing – review & editing. H-JJ: Data curation, Writing – review & editing. ISJ: Data curation, Writing – review & editing. BK: Conceptualization, Data curation, Formal analysis, Funding acquisition, Investigation, Methodology, Supervision, Writing – original draft, Writing – review & editing.
